# *Graptopetalum Paraguayense* Ameliorates Chemical-Induced Rat Hepatic Fibrosis *In Vivo* and Inactivates Stellate Cells and Kupffer Cells *In Vitro*

**DOI:** 10.1371/journal.pone.0053988

**Published:** 2013-01-15

**Authors:** Li-Jen Su, Chia-Chuan Chang, Chih-Hsueh Yang, Shur-Jong Hsieh, Yi-Chin Wu, Jin-Mei Lai, Tzu-Ling Tseng, Chi-Ying F. Huang, Shih-Lan Hsu

**Affiliations:** 1 Institute of Systems Biology and Bioinformatics, National Central University, Jhongli City, Taiwan; 2 Division of Medicinal Chemistry, National Research Institute of Chinese Medicine, Taipei City, Taiwan; 3 Department of Education and Research, Taichung Veterans General Hospital, Taichung City, Taiwan; 4 Department of Life Science, Fu Jen Catholic University, Taipei County, Taiwan; 5 Biomarker Technology Development Division, Biomedical Technology and Device Research Labs, Industrial Technology Research Institute, Hsinchu, Taiwan; 6 Institute of Biopharmaceutical Sciences, National Yang-Ming University, Taipei, Taiwan; IIT Research Institute, United States of America

## Abstract

**Background:**

*Graptopetalum paraguayense* (GP) is a folk herbal medicine with hepatoprotective effects that is used in Taiwan. The aim of this study was to evaluate the hepatoprotective and antifibrotic effects of GP on experimental hepatic fibrosis in both dimethylnitrosamine (DMN)- and carbon tetrachloride (CCl_4_)-induced liver injury rats.

**Methods:**

Hepatic fibrosis-induced rats were fed with the methanolic extract of GP (MGP) by oral administration every day. Immunohistochemistry, biochemical assays, and Western blot analysis were performed. The effects of MGP on the expression of fibrotic markers and cytokines in the primary cultured hepatic stellate cells (HSCs) and Kupffer cells, respectively, were evaluated.

**Results:**

Oral administration of MGP significantly alleviated DMN- or CCl_4_-induced liver inflammation and fibrosis. High levels of alanine transaminase, aspartate transaminase, bilirubin, prothrombin activity and mortality rates also decreased in rats treated with MGP. There were significantly decreased hydroxyproline levels in therapeutic rats compared with those of the liver-damaged rats. Collagen I and alpha smooth muscle actin (α-SMA) expression were all reduced by incubation with MGP in primary cultured rat HSCs. Furthermore, MGP induced apoptotic cell death in activated HSCs. MGP also suppressed lipopolysaccharide-stimulated rat Kupffer cell activation by decreasing nitric oxide, tumor necrosis factor-α and interleukin-6 production, and increasing interleukin-10 expression.

**Conclusions:**

The results show that the administration of MGP attenuated toxin-induced hepatic damage and fibrosis *in vivo* and inhibited HSC and Kupffer cell activation *in vitro*, suggesting that MGP might be a promising complementary or alternative therapeutic agent for liver inflammation and fibrosis.

## Introduction

Hepatic fibrosis, a precursor of cirrhosis, is characterized by an excessive generation of extracellular matrix constituents (particularly collagens) that impair normal function with progression of liver disease to cirrhosis [1]. Liver fibrosis can result in the development of liver carcinoma and the mortality of patients with liver fibrosis is gradually increasing [2]. Numerous agents, including corticosteroids, penicillamine, methotrexate, silymarin and colchicines, have been used in the therapy of hepatic fibrosis, but there is no definitive treatment [3], [4], [5], [6]. An effective therapy for treatment of patients with hepatic fibrosis is thus urgently needed.

Herbal medicines that have been used in China for thousands of years are now being manufactured in China as drugs with standardized quality and quantity of ingredients. Over the past several decades, there has been a growing trend in Western countries to use herbal medicines to treat a wide range of diseases, including obesity, insomnia, eczema, arthritis, immunodeficiency syndrome, inflammatory diseases, and chronic liver diseases [7]. *Graptopetalum paraguayense* (GP) is a Crassulacean acid metabolism plant with antioxidative and antiproliferative activities [8], [9] and is commonly used as a health food in Taiwan. It is considered to have potentially beneficial effects in hypertension, diabetes, hyperuricemia, and chronic liver diseases [10]. However, there is no definitive experimental or clinical evidence for the efficacy of GP in the treatment of these diseases.

Our previous study indicated that a concentration of 70% methanolic extract of GP (MGP) was the most effective for liver protection. The present study was initiated to examine the effects of MGP on hepatic fibrosis *in vivo* through induction by dimethylnitrosamine (DMN) and carbon tetrachloride (CCl_4_) in rats. The effects of MGP on the myofibroblast transformation of rat hepatic stellate cells (HSCs) and Kupffer cell activation in primary culture were also investigated. The results indicate that the oral administration of MGP attenuated collagen deposition in toxin-induced liver fibrosis in rats. MGP also inhibited the proliferation of activated HSCs and reduced collagen and alpha smooth muscle actin (α-SMA) expression. Moreover, MGP was shown to be capable of modulating lipopolysaccharide (LPS)-stimulated tumor necrosis factor alpha (TNF-α), interleukin 6 (IL-6), interleukin 10 (IL-10), and nitric oxide (NO) production in Kupffer cells.

Our findings indicate that the protective effects of MGP against liver injury likely involve multiple mechanisms, including an anti-inflammatory effect through decreased TNF-α, IL-6 and NO production in LPS-stimulated conditions as well as an antifibrogenic effect that is mediated by inhibiting the activation of cell transformation and by the induction of apoptosis in HSCs. These observations strongly suggest that MGP might have therapeutic potential for treatment of liver fibrosis and chronic liver disorders.

## Materials and Methods

### Ethics Statement

All animal work has been conducted according to relevant national and international guidelines. The animal use protocol has been reviewed and approved by the Institutional Animal Care and Use Committee (IACUC) of Taichung Veterans General Hospital. IACUC Approval Number is La-97475. Period of Protocol was valid from: 08/01/2008 to 07/31/2009. The Principle Investigator (PI) is Shih-Lan Hsu.

### Preparation of MGP

The leaves of GP were purchased from a local herb farm in Taiwan and were washed with distilled water, air-dried overnight, and then freeze-dried at −50°C with a freeze dryer and ground to a powder (100 mesh). Lyophilized GP powder was stored at 4°C until use. The method for preparing MGP was modified based on the procedure described by Wang et al. [11] MGP extract was prepared as follows: lyophilized GP powder (100 g) was dissolved in 70% methanol (1000 ml). After centrifugation at 1400×g for 20 min, the resulting precipitates were discarded and the supernatant was filtered through a 0.22 µm filter. The filtrate was evaporated to dryness on a rotary evaporator and then lyophilized. The dried methanolic extract powder of GP was stored at 4°C until use.

### Preparation of Liver Injury and Fibrosis Model Rats by DMN and CCl_4_

Male Sprague-Dawley rats, weighing 250 to 300 g, were purchased from the National Laboratory Animal Breeding and Research Center, National Science Council, Taiwan. All experiments were performed in accordance with The National Laboratory Animal Breeding and Research Center’s guidelines. Liver injury and fibrosis model rats were produced by the administration of DMN (Sigma, USA) as previously reported [12] with minor modification. For the DMN-induced injury model, rats were intraperitoneally (i.p.) injected with DMN diluted in phosphate-buffered saline (PBS) (7 mg/kg per day) on the first 3 days each week for 3 weeks to cause liver injury and fibrosis. The control groups were injected with PBS alone. Fifty-four rats were randomized into three experimental groups (n = 18 in each group) as follows: (1) Control (injection of PBS i.p. and oral administration of water); (2) DMN (injection of DMN i.p. and oral administration of water); (3) DMN+MGP (injection of DMN i.p. and oral administration of MGP). MGP was suspended in water and administered orally once each day at a dose of 400 mg/kg for 6 weeks, starting on the 8^th^ day after the first injection of DMN. [The 8^th^ day means that there was a 7-day period after the first DMN injection and on the following day, i.e., the 8^th^ day, MGP was given.] Control rats received distilled water alone.

For the CCl_4_-induced liver injury model, rats were randomly sorted into three groups (n = 20): control, model group (CCl_4_), and CCl_4_+MGP group. Each group, except the control group, received an oral dose of CCl_4_ (1 ml/kg body weight) (Panreac Quimica SAU, Spain) twice a week (40%, diluted in olive oil) for 10 weeks [13]. MGP was suspended in water and was administered orally once per day at a dose of 400 mg/kg for 9 weeks, starting on the 8^th^ day after the first treatment of CCl_4_. Control rats received only distilled water. At the end of the *in vivo* experiments, blood samples were taken to determine biochemical indicators. The livers were excised, weighed and fixed in formaldehyde for histopathological examination. Protocols for these studies were reviewed by the Ethics Committee on Animal Experimentation of Taichung Veterans General Hospital.

### CCl_4_-induced Acute Hepatotoxicity Model

SD rats were intraperitoneally injected with a single-dose of CCl_4_ (2 ml/kg in olive oil) to induce acute hepatotoxicity and were orally fed with 400 mg/kg MGP daily, rats were sacrificed after 4 days. There were three experimental groups of rat (n = 6 per group), namely: control rat (vehicle only), CCl_4_-treated rat and CCl_4_+MGP treated rat (400 mg/kg MGP).

### Histopathological and Biochemical Examination of the Liver

MGP was orally administered to toxin-treated rats and the effects on liver fibrosis were evaluated by histopathological examinations of hepatic fibrosis with hematoxylin/eosin staining, Sirius red-Fast green staining, and computerized score of hepatic fibrosis. Liver specimens were fixed with phosphate-buffered formaldehyde, embedded in paraffin, and stained with hematoxylin-eosin. The fibrosis scoring system was modified from the Histology Activity Index (HAI) [14], [15], includes necroinflammatory and fibrosis as previously described [16]. Three represented images of each histology sample section (at 100× magnification) of each rat have been selected randomly and scored. The differential staining of collagenous and noncollagenous proteins was performed with 0.1% Sirius red and 0.1% Fast green as a counter-stain in saturated picric acid. In this procedure, collagen is stained red [17]. Liver tissue sections were photographed using AXIO, Imager. A1 microscope (ZEISS, Germany). The content of collagen fibers was estimated by Image-Pro Plus 5.0. Biochemical determinations of hydroxyproline content in liver and serum biochemical markers including alanine transaminase (AST), aspartate transaminase (ALT), bilirubin, albumin, prothrombin time, and platelet number were also performed using commercial kits (Wako Inc., Japan).

### Immunocytochemical Staining for Collagen I and α-SMA

To detect collagen I and α-SMA in cultured HSCs, immunocytochemical staining was performed using anti-rat collagen I and α-SMA antibodies (Calbiochem-Merck, USA) and a secondary fluorescein isothiocyanate (FITC)-conjugated goat anti-mouse IgG (Santa Cruz Biotechnology, USA). Photomicrographs were taken using a fluorescence microscope.

### Immunohistochemical Staining for TNF-α and IL-6

Hepatic staining for TNF-α and IL-6 was examined by immunohistochemical procedure. Briefly, the sections were deparaffinized and treated with 3% hydrogen peroxide to inactivate endogenous peroxidases. Epitope unmasking was performed by immersing sections in antigen retrieval solution A (BD Pharmingen, USA) and heating to 120°C for 20 min. After cooling, blocking with 10% fetal bovine serum at room temperature for 30 min followed by the sequential application of mouse anti-rat TNF-α and IL-6 antibodies, biotin-conjugated goat anti-mouse immunoglobulin G, and streptavidin-conjugated horseradish peroxidase for 30 min. Finally, the sections were incubated in diaminobenzidine according to the manufacturer’s instructions (Vector Laboratories, USA) and counter-stained with hematoxylin.

### Determination of Hydroxyproline Content

Livers were collected at the moment of sacrifice, and 150 mg of liver tissue was subjected to acid hydrolysis to determine the amount of hydroxyproline according to a procedure described elsewhere [18] with some modifications. Briefly, 2 ml of homogenized liver tissue was hydrolyzed by adding 3 ml of 10 N HCl and then incubated at 110°C for 16 h. After cooling, the hydrolysate was filtered through a 0.45 µm filter. A mixture of 1 ml of 6 N NaOH and 1 ml of citrate buffer was added to 1 ml hydrolysate and mixed well, and then, 1 ml of 0.05 M chloramine-T in citrate-acetate buffer (pH 6.0–6.5) in the presence of *n*-propanol (1.32 M) was added to the mixture. The solution was thoroughly mixed and incubated for 20 min at room temperature. The reaction was then stopped by adding 1 ml perchloric acid/*p*-dimethylaminobenzaldehyde solution in *n*-propanol (32.5 ml 70% perchloric acid, 18.75 g dimethylaminobenzaldehyde, 75 ml *n*-propanol) and then incubated at 60°C for 30 min. After cooling, the absorbance was determined by a spectrophotometer at 550 nm. The concentration of hydroxyproline was calculated from standard curves using pure hydroxyproline as a standard. The results are expressed as micrograms of hydroxyproline per gram of liver tissue.

### Isolation of HSCs and Cell Viability Determination

HSCs were isolated from the liver of male Sprague-Dawley rats as described previously [19] with a minor modification. Briefly, for each rat, the liver was perfused *in situ* through the portal vein with a 16-gauge cannula, first with Ca^2+^/Mg^2+^-free HBSS solution at 37°C for 10 min at a flow rate of 10 ml/min, followed by 0.1% pronase E (Merck, Darmstadt, Germany) in HBSS solution for 10 min, and then with 0.3% collagenase (Wako, Osaka, Japan) in HBSS solution for 30 min. The digested liver was excised, minced with scissors, and incubated in HBSS solution containing 0.05% pronase E and 20 µg/ml DNase for 30 min. The resulting suspension was filtered through nylon mesh (150 mm in diameter). An HSC-enriched fraction was obtained by centrifugation of the filtrate in an 8.2% Nycodenz (Nycomed, Norway) solution at 1,400×g at 4°C for 20 min. The cells in the upper layer were washed by centrifugation at 450×g at 4°C for 10 min and suspended in DMEM supplemented with 10% fetal bovine serum, 100 U/ml penicillin, 100 µg/ml streptomycin, and 1% L-glutamine. The purity of the isolated HSC was assessed through direct cell counting under phase-contrast microscopy by intrinsic vitamin A autofluorescence and by immunohistochemistry using a monoclonal antibody against desmin (DAKO; diluted 1∶40). Cell viability was examined by trypan blue dye exclusion. Both cell purity and viability were in excess of 90%. HSCs were plated at a density of 5×10^5^ cells per well in 1 ml of culture medium on culture dishes, and the culture medium was changed 2 days after plating. Cells were maintained at 37°C in a 5% CO_2_ incubator for the indicated time points.

### Isolation of Kupffer Cells

Rat Kupffer cells were isolated from the liver of male Sprague-Dawley rats as described previously [20] with a minor modification. Briefly, rats were fasted overnight, and the livers were perfused through the portal vein with perfusion buffer (Ca^+2^ and Mg^+2^-free Krebs-Henseleit solution containing 0.2% glucose, 0.2% bovine serum albumin, 0.03% collagenase, and 0.02% protease). The cell suspension was filtered through two layers of nylon mesh, and the filtrate was centrifuged at 600×g for 45 s. The supernatant was then centrifuged at 900×g for 6 min. The pellet was suspended in Gey’s balanced salt solution (GBSS) without NaCl containing 17.5% metrizamide. One milliliter of GBSS was layered over 6 ml of the cell suspension after centrifugation at 1400×g for 20 min. The interface layer containing Kupffer cells was isolated, washed in PBS, and then suspended in William’s medium E containing 10% fetal calf serum. Further purification of the cells was achieved by attachment to the plastic plates for 2–3 h. Medium was renewed after 3 h and on the following day. Cells were then used on the third day. Kupffer cells were identified by their ability to phagocytose 1 µm latex particles and by their staining with ED2. Cells isolated in this way were 90% Kupffer cells and less than 95% viable (Trypan Blue exclusion).

### TUNEL Assay

The TUNEL assay was performed according to the manufacturer’s protocol (Boehringer Mannheim, Roche). Briefly, rat activated HSCs were treated without or with MGP (400 µg/ml) for 72 h. After incubation, cells were fixed with 4% paraformaldehyde, incubated in reaction buffer (34 mU/ml terminal transferase, 280 pmol of dATP, 90 pmol of fluorescein-11-dUTP, 30 mM Tris-HCl, 140 mM sodium cacodylate, 1 mM CaCl_2_, pH = 7.2) at 37°C for 60 min in the dark, and then rinsed twice with PBS. Cells were visualized using a fluorescence microscope. TUNEL positive cells were counted as apoptotic.

### Caspase Activity Assay

Caspase-2, caspase-3, caspase-8 and caspase-9 activities were estimated according to the manufacturer’s protocol. Briefly, cell lysates (100 µg total protein) were added to reaction mixtures (final volume 50 µL) containing fluorogenic peptide substrate specific for each caspase. The reaction was performed at 37°C for 2 h. Fluorescence intensity was measured with a fluorescence microplate reader (Thermo Labsystem, Finland) (excitation wavelength 400 nm, emission wavelength 505 nm). Caspase-2 inhibitor (Z-VDVAD-fmk), caspase-3 inhibitor (Z-DEVD-fmk), caspase-8 inhibitor (Z-IETD-fmk), and caspase-9 inhibitor (Z-LEHD-fmk) were obtained from Kamiya (Thousand Oaks, USA).

### Protein Preparation and Western Blot Analysis

HSCs were treated without or with 400 µg/ml MGP for 48 hrs. After treatment, cells were lysed, and total protein was isolated. The protein content was determined by the Bradford method. For Western blot analysis, equal amounts of total protein were loaded onto SDS-polyacrylamide gels, and the proteins were electrophoretically transferred onto a PVDF membrane (Millipore, Bedford, MA). The protein expression was detected by each immunoblotting with the corresponding specific primary antibodies (against collagen I, α-SMA, B-cell CLL/lymphoma 2 (Bcl-2), myeloid cell leukemia sequence 1 (Mcl-1), Bcl2-associated X protein (Bax), and β-actin) at room temperature for 4 hrs. After washing three times with TBST, the membrane was incubated with horseradish peroxidase-labeled secondary antibody for 1 hr. The membrane was washed, and detection was performed using the enhanced chemiluminescence blotting detection system (Amersham, USA).

### TNF-α and IL-6 Measurement

The concentrations of TNF-α and IL-6 in the medium were determined by ELISA kits OptEIA Set (BD Biosciences, USA) that were specific against rat cytokines. Assays were performed according to the manufacturer’s instructions.

### Nitrite Measurement

The nitrite concentration in the medium was measured using the Griess reaction, and the calculated concentration was taken as an indicator of NO production. The medium of cell cultures was harvested and mixed with an equal volume of Griess reagent (1% sulfanilamide in 5% phosphoric acid and 0.1% naphthyl ethylenediamine dihydrochloride in water). The optical density at 550 nm (A_550_) was measured and calculated against a sodium nitrite standard curve.

### Statistical Analysis

All data are shown as the mean±S.D. from at least 3 independent experiments. Figures were obtained from at least three independent experiments with similar patterns. The biochemical experimental data were analyzed by two-way ANOVA. When the interaction was significant, Tukey’s multiple-comparison test was performed. Survival analyses were conducted using the Kaplan-Meier method. Comparisons were made using one-way ANOVA followed by the Mann-Whitney test. All analyses were performed with SPSS software version 13.0 (SPSS Inc., USA).

## Results

### Effects of GP Methanolic Extract on Liver Fibrosis in Rats given DMN

**Figure 1:**
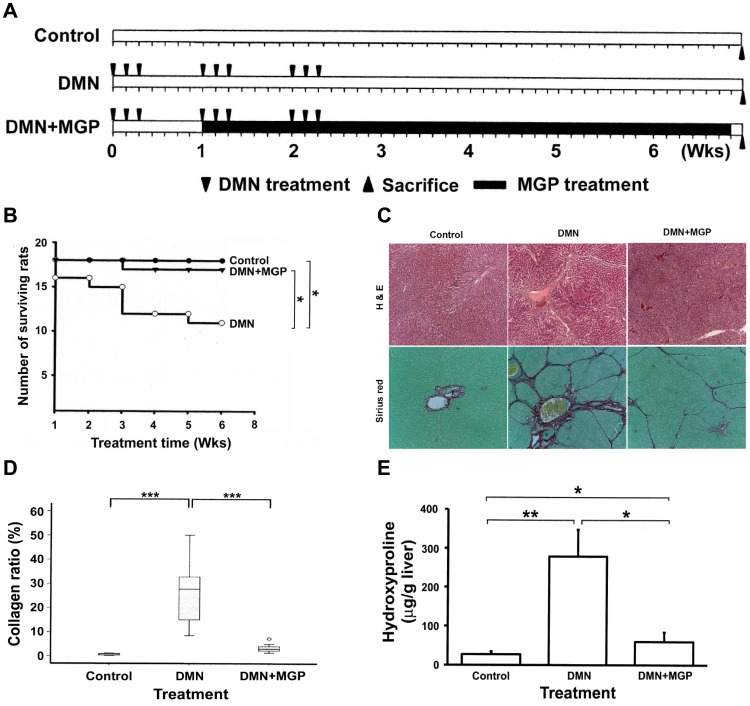
Effects of MGP on liver fibrosis in rats following DMN administration. (**A**) Schematic illustration for *in vivo* experiments. Each Sprague-Dawley rat was either injected with DMN three times per week for three consecutive weeks (triangle) or injected with normal saline as a control under the same regime. Subsequently, MGP (400 µg/ml) was suspended in water and administered orally from day 8 until day 49 (black column). Rats were weighed and sacrificed 7 weeks after first DMN injection. (**B**) Survival analysis. The death of rats was recorded every week and used to calculate the survival rate. Livers were excised and fixed in formaldehyde for (**C**) histopathological examinations. The rat liver sections were stained with hematoxylin-eosin and Sirius red-Fast green. The original magnification was 40×. (**D)** Collagen content. The collagen areas were calculated in eight fields independently, and the extent of fibrosis in each field was expressed as the area of collagen as a percentage of the total area of the field. There was a significant difference between the DMN and the DMN+GP groups. (**E**) Hydroxyproline content. The level of hydroxyproline in the liver tissue was measured as described in the Materials and Methods section. Significance under *p*-values<0.05, 0.001 and 0.0001 are marked with *, ** and ***, respectively.

Repeated administration of DMN or CCl_4_ to rats resulted in a reproducible animal model of liver fibrosis [21], [22] and enabled us to examine the hepatoprotective effects of MGP on DMN- and CCl_4_-induced chronic liver injury in rats. The schematic illustration for *in vivo* DMN-induced hepatic fibrosis experiments is shown in Fig. 1A. After 6 weeks of treatment, the liver damage and fibrosis-improving effects of MGP were assessed according to changes in body, liver and spleen weights, the levels of ALT, AST, bilirubin, and albumin, prothrombin activity, and platelet number, as well as liver hydroxyproline content and fibrosis area. As shown in Table 1, treatment with DMN caused a significant decrease in rat body and liver weights, and increased spleen weight compared with those of the control rats. By comparison, oral feeding of MGP significantly increased body and liver weights and decreased spleen weight. Moreover, the survival rates in the DMN-treated and DMN-MGP-treated groups were 61.1% (11 of 18 rats survived) and 94.4% (17 of 18 rats survived), respectively. No rats died in the control group (Fig. 1B).

**Table 1:** Body, liver, and spleen weights in DMN- and/or MGP-treated rats.

		Weight (g)		
Group	n	Body	Liver	Spleen
Control	18	543.0±47.4^c^	26.6±1.2^c^	1.0±0.0^a^
DMN	11	473.8±39.0^a^	17.8±3.2^a^	2.6±0.4^b^
DMN+MGP	17	513.1±39.1^b^	22.2±2.2^b^	1.2±0.2^a^

a n: number of surviving rats.

b Data expressed as mean±S.D.

c Values in the same column with different superscripts are significantly different (*p*<0.05).

Serum biochemical marker determinations showed that the serum AST, ALT and bilirubin levels and the prothrombin time increased, whereas the serum albumin and platelet levels decreased in DMN-treated rats compared with those of the normal controls (Table 2). However, oral feeding with MGP effectively reduced DMN-induced elevation of AST, ALT, bilirubin, and prothrombin time. DMN-mediated decline in serum albumin and platelet number was increased by MGP administration.

**Table 2:** Effect of administration of MGP on serum biochemical indicators in DMN-induced liver injury rats.

	AST	ALT	PT	PLT	Bilirubin	Albumin
Group	(U/L)	(U/L)	(sec)	(10^3^/uL)	(µmole/L)	(g/dL)
Control	94.2±15.6^a^	39.1±6.5^a^	13.0±1.2^a^	894.9±157.4^b^	0.1±0.0^a^	4.4±0.3^b^
DMN	520.8±198.8^b^	224.7±47.8^c^	18.3±6.1^b^	269.6±183.4^a^	1.0±0.4^b^	2.8±0.9^a^
DMN+MGP	129.1±43.5^a^	67.2±18.4^b^	13.4±1.0^a^	785.9±96.0^b^	0.1±0.1^a^	4.3±0.5^b^

a Data expressed as mean±S.D.

b Values in the same column with different superscripts are significantly different (*p*<0.05).

c AST: aspartate transaminase; ALT: alanine transaminase; PT: prothrombin time; PLT: number of platelets.

The effect of MGP on liver fibrogenesis was investigated by hematoxylin-eosin staining. We found that administration of DMN induced centrilobular and periportal deposition of fibers, and continuous fibrotic septa were observed between the central and portal veins (Fig. 1C, upper panel). Treatment with MGP thus obviously suppressed fibril deposition. Data from Fast green-Sirius red staining exhibited a gradual and marked increase in collagen fiber content in the livers of DMN-treated rats, displaying bundles of collagen (stained in red) surrounding the lobules. This resulted in large fibrous septa, indicating the onset of severe fibrosis (Fig. 1C, lower panel). However, the thickening of these collagen bundles was reduced drastically in the DMN+MGP group. Furthermore, there were very low levels of collagen deposition in the livers of control rats (lower panel of Fig. 1C).

The content of collagen in liver tissue was measured by computer-assisted morphometric analysis of liver sections. The results indicated that DMN-induced fibrotic livers had higher levels of collagen than those of the control rat livers. In rats fed with MGP after administration of DMN, collagen levels were decreased significantly (Fig. 1D). Next, hepatic collagen was also quantified by the measurement of hepatic hydroxyproline content, which parallels the extent of fibrosis. Similar results were found in the DMN group which had significantly higher hydroxyproline content than that of the DMN+MGP group (DMN, 276±67 µg/g *vs.* DMN+MGP, 60±26 µg/g, *p*<0.01) and the control group (29±5 µg/g, *p*<0.001) (Fig. 1E).

### Effects of GP Methanolic Extract on CCl_4_-induced Liver Fibrosis in Rats

**Figure 2:**
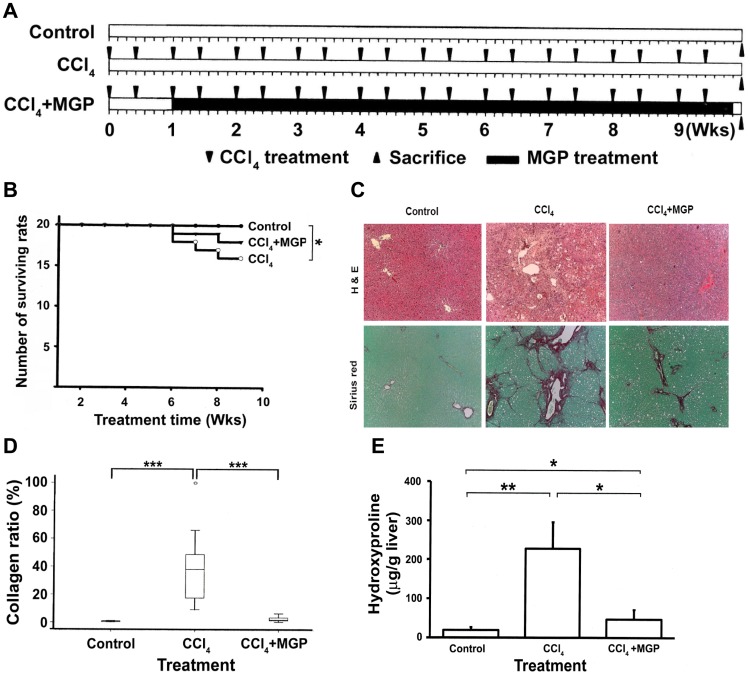
Effect of MGP on liver fibrosis induced by CCl_4_ in rats. (**A**) Schematic illustration for *in vivo* experiments. Each group except the control received an oral dose of CCl_4_ (1 ml/kg body weight) twice a week (40%, diluted in olive oil) for 10 weeks (triangle). The control group was injected with normal saline. GP extract was suspended in water and administered orally once per day at a dose of 400 mg/kg for 9 weeks (black column), starting 7 days after the first treatment with CCl_4_. Control rats received distilled water alone. Rats were weighed and sacrificed 10 weeks after the first CCl_4_ treatment. (**B**) Survival analysis. The death of rats was recorded every week and used to calculate the survival rate. Livers were excised and fixed in formaldehyde for (**C**) histopathological examination. The rat liver sections were stained with hematoxylin-eosin and Sirius red-Fast green. The original magnification was 40×. (**D**) Collagen content. The collagen areas were calculated in eight fields independently and the extent of fibrosis in each field was expressed as the area of collagen as a percentage of the total area of the field. (**E**) Hydroxyproline content. The level of hydroxyproline in the liver tissue was measured as described in the Materials and Methods section. Significance under *p*-values<0.05, 0.001 and 0.0001 were marked with *, ** and ***, respectively.

The CCl_4_-induced liver injury and fibrosis experimental schedule is presented in Fig. 2A, where the CCl_4_ was repeatedly administered (1 ml/kg, 2 times per week) and orally fed to the rats with or without MGP for 9 weeks. All of the rats in the control group survived during the experimental period. The survival rate of the CCl_4_-treated group and the CCl_4_-MGP-treated group was 80% (16 of 20 rats survived) and 90% (18 of 20 rats survived), respectively (Fig. 2B). The influences of CCl_4_ and MGP on rat body, liver, and spleen weights are depicted in Table 3. As can be seen, the body weight of CCl_4_-administered rats significantly decreased, but treatment with MGP exhibited a significant increase in body weight when compared with that of the CCl_4_-administered group. MGP also reversed CCl_4_-induced splenomegaly.

**Table 3:** Body, liver, and spleen weights in CCl4- and/or MGP-treated rats.

		Weight (g)		
Group	n	Body	Liver	Spleen
Control	20	691±50^b^	31±6.0	1.1±0.5^ab^
CCl_4_	16	651±71^a^	30±7.7	1.4±0.5^b^
CCl_4_+MGP	18	679±64^ab^	29±5.0	1.0±0.3^a^

a n: number of surviving rats.

b Data expressed as mean±S.D.

c Values in the same column with different superscripts are significantly different (*p*<0.05).

As shown in Table 4, the serum AST, ALT and bilirubin levels as well as the prothrombin time were increased, whereas the albumin and platelet levels decreased in CCl_4_-treated rats. Oral administration of MGP significantly reduced the serum levels of AST, ALT, and bilirubin while the serum level of albumin increased. In addition, treatment with CCl_4_ drastically reduced serum platelet number and prolonged the prothrombin time. Coadministration of MGP dramatically reversed the CCl_4_-induced platelet number decrease and prothrombin time elongation.

**Table 4:** Effect of administration of MGP on serum biochemical indicators in CCl4-induced liver injury rats.

	AST	ALT	PT	PLT	Bilirubin	Albumin
Group	(U/L)	(U/L)	(sec)	(10^3^/uL)	(µmole/L)	(g/dL)
Control	94.9±27^a^	45.9±19^a^	13.6±0.7^a^	912±167^b^	0.1±0.01^a^	5.0±0.5^b^
CCl_4_	314±48^c^	282±53^c^	15.3±0.9^b^	767±141^a^	1.1±0.50^b^	3.8±0.3^a^
CCl_4_+MGP	180±59^b^	132±10^b^	13.0±0.7^a^	1047±203^c^	0.1±0.05^a^	4.9±0.1^b^

a Data expressed as mean±S.D.

b Values in the same column with different superscripts are significantly different (*p*<0.05).

c AST: aspartate transaminase; ALT: alanine transaminase; PT: prothrombin time; PLT: number of platelets.

Representative photographs of liver morphology examined by hematoxylin-eosin staining are shown in Fig. 2C. Compared with normal rat liver morphology, CCl_4_-treated rat livers showed diffuse fatty tissue and micronodular fibrosis along the central vein and portal area. However, feeding CCl_4_-injured rats with MGP significantly ameliorated CCl_4_-induced fibrosis and collagen accumulation. In addition, the degree of CCl_4_-induced fatty liver and other pathological changes were also mitigated (Fig. 2C, upper panel). Fast green-Sirius red staining results demonstrated that little red staining was observed in the pericentral area in the control rat livers. In contrast, the livers injured by chronic CCl_4_ treatment displayed an excessive deposition of collagen fibrils. However, MGP treatment clearly suppressed collagen accumulation (Fig. 2C, lower panel). Quantitative analysis of Fast Green-Sirius red staining photographs by image analysis showed that hepatic collagen was increased 11-fold as a result of CCl_4_ exposure and that MGP treatment decreased CCl_4_-mediated elevation of hepatic collagen by approximately 80% (Fig. 2D).

The biochemical determination of hydroxyproline strongly correlated with the diminished CCl_4_-induced fibrosis by MGP, as measured by image morphometric analysis (Fig. 2D and 2E). These data suggest that MGP could suppress hepatic fibrosis in chronic liver injury. Collectively, our findings indicate that MGP effectively alleviated fibrogenic progression in the liver following toxic chemical (both DMN and CCl_4_) administration.

Data from HAI pathological fibrosis scoring analysis indicated that treatment with MGP could significantly reduce DMN- and CCl_4_-induced hepatic fibrosis (Table 5). Based on our results, DMN indeed caused more severe liver damage than CCl_4_. DMN caused cirrhosis whereas CCl_4_ just caused fibrosis. We also found that DMN induced an irreversible liver fibrosis and cirrhosis model while CCl_4_ induced a reversible liver fibrosis model.

**Table 5:** The summary of histopathological fibrosis score for the rat models by modified Hepatitis Activity Index (HAI) system.

	DMN model (6^th^ week)			CCl_4_ model (9^th^ week)		
Fibrosis score	Control n (%)	DMN n (%)	DMN+MGP n (%)	Control n (%)	CCl_4_ n (%)	CCl_4_+MGP n (%)
0–1	18 (100)	3 (19)	12 (67)	20 (100)	7 (35)	16 (80)
2–3	0 (0)	13 (81)	6 (33)	0 (0)	13 (65)	4 (20)

Fibrosis was graded as (0–1) for observations ranging from normal tissue to fibrous expansion of portal tracts and (2–3) for bridge fibrosis to frequent bridging fibrosis with nodule formation. The number of rats was counted and used to calculate the percentage of each histopathological level at each time point.

### MGP Suppresses the Activation of Primary Cultured HSCs

**Figure 3:**
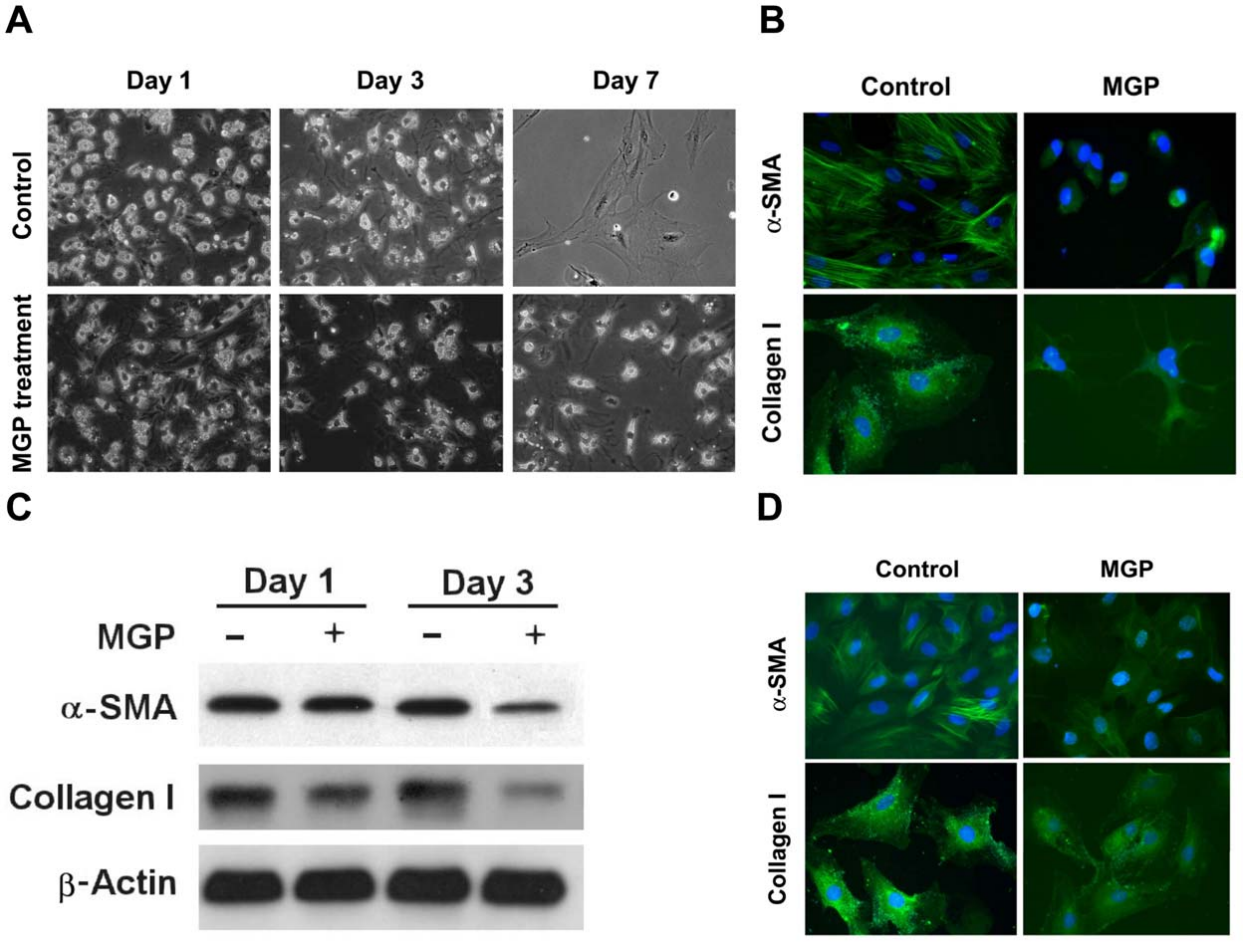
MGP inhibits transformed activation of HSCs. Isolated HSCs were incubated in the presence or absence of 200 µg of MGP for 1, 3, and 7 days. After treatment, (**A**) the cell morphology was investigated by phase-contrast microscopy, and (**B**) the expression levels of α-SMA and collagen I after 7 days incubation were examined by immunostaining. (**C**) MGP suppressed α-SMA and collagen I expression in activated HSCs. The activated HSCs (5 passages) were incubated without or with 200 µg of MGP for 1 and 3 days. After incubation, the expression levels of α-SMA and collagen I were examined by immunoblotting. (**D**) The expression of α-SMA and collagen I after 3 days incubation was investigated by immunostaining.

The activation of HSCs is the key process in the pathogenesis of hepatic fibrosis, as activated HSCs are transformed into the myofibroblastic phenotype expressing myogenic marker α-SMA and then differentiate into collagen type I-producing cells. To gain insight into the cellular and molecular mechanisms that are involved in the regulation of fibrosis reversion induced by MGP, we examined the direct effects of MGP on HSC activation. For this study, the primary cultured HSCs were isolated from rats and then incubated with 200 µg/ml MGP for 1, 3, and 7 days. The morphological changes and the expression of α-SMA and collagen I (markers of HSC activation) were then investigated. As shown in Fig. 3A, the isolated HSCs were transformed to myofibroblast-like cells with enlarged cell bodies and exhibited immunoreactive α-SMA and collagen I after 7 days of incubation (Fig. 3B). The addition of MGP prevented HSC myofibroblast-like activation (Fig. 3A) and largely suppressed the immunoreactivity of α-SMA and collagen I (Fig. 3B).

To test whether MGP reversed the myofibroblastic-like activation of transformed HSCs, activatedHSCs (over 5 passages) were treated without or with 200 µg/ml MGP. The expression of myogenic markers α-SMA and collagen I was then examined. Data from Western blotting showed that a large amount of α-SMA and collagen I were expressed in the activated HSCs while a significantly reduced expression of these two activated HSC markers was observed after exposure to MGP (Fig. 3C). Moreover, the immunoreactivity of both α-SMA and collagen I was strong in activated HSCs, but treatment with 200 µg/ml MGP for 3 days largely inhibited the expression of these two markers. These observations indicate that MGP suppressed activation of HSC transformation and inhibited α-SMA and collagen I production in primary cultured HSCs.

### MGP Induces Apoptosis of Activated HSCs

**Figure 4:**
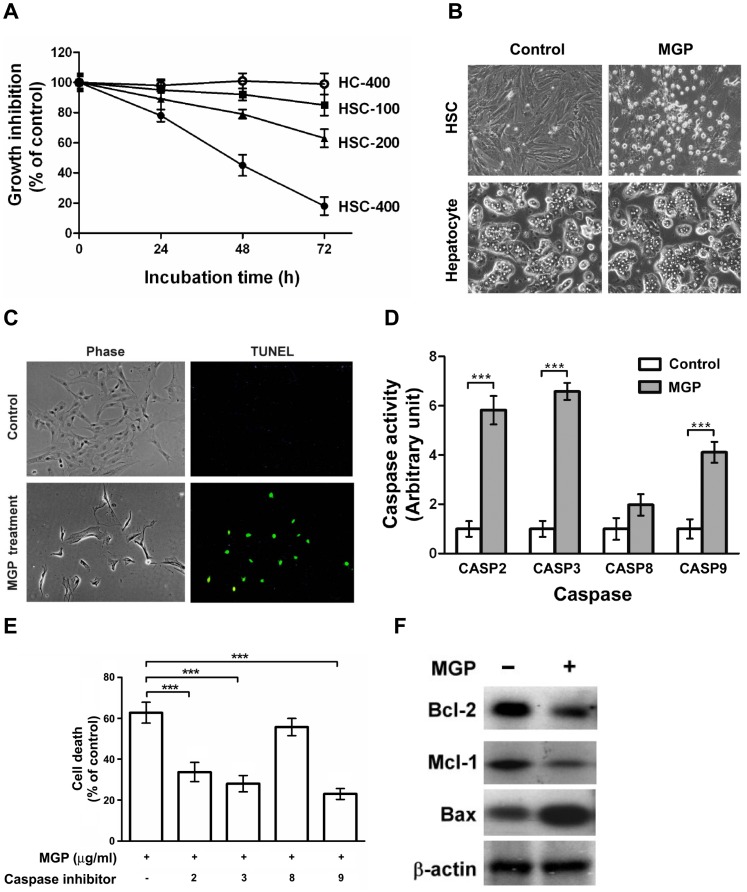
MGP induces apoptotic death in activated HSCs. (**A**) Cytotoxic effect of MGP. Activated HSCs were treated with various concentrations of MGP (100, 200, and 400 µg/ml) for 24, 48, and 72 h. Viable cells were measured by the trypan blue dye exclusion method. (**B**) Morphological changes. HSCs and hepatocytes were incubated with MGP (400 µg/ml) for 72 h. Cell morphology was investigated under an Olympus IX70 phase-contrast microscope. (**C**) Induction of apoptosis by MGP. The HSCs were maintained in the presence or absence of MGP (400 µg/ml) for 48 h. After treatment, the TUNEL assay was performed as described in the Materials and Methods. The morphology of HSCs was again investigated under a phase-contrast microscope. TUNEL positive cells were considered apoptotic cells. The original magnification was 200×. (**D**) Activation of caspases by MGP. Activated HSCs were treated without or with MGP (400 µg/ml) for 48 h and the activity of each caspase was then assessed. (**E**) Inhibitors of caspases protected against MGP-induced apoptosis. Activated HSCs were pretreated with 100 µg/ml caspase inhibitor for 1 h and then treated with MGP (400 µg/ml) for another 72 h. After treatment, apoptotic cell count was assessed by the TUNEL assay. Significance for *p*-value<0.0001 is marked with ***. (**F**) Regulation of anti-apoptotic and proapoptotic molecules by MGP. Activated HSCs were treated without or with MGP (400 µg/ml) for 48 h. The levels of Bcl-2, Mcl-1, and Bax were determined by Western blot analysis.

We also assayed the effects of MGP on the viability of activated HSCs (over 5 passages) and primary cultured hepatocytes. As shown in Fig. 4A, MGP caused a dose-dependent reduction in viability of activated HSCs within 72 h. Conversely, the viability of primary cultured rat hepatocytes did not alter after 72 h of MGP (400 µg/ml) incubation. Morphological investigation of MGP-treated HSCs showed apoptotic features (Fig. 4B). We therefore determined whether apoptosis was a mechanism in MGP-mediated reduced viability of HSCs. The TUNEL assay confirmed substantial genomic DNA fragmentation upon incubation with MGP (400 µg/ml) for 48 h (Fig. 4C). However, at the same dose of MGP, no TUNEL positive cells could be detected in hepatocyte cultures.

To determine the role of caspases in MGP-induced apoptosis, the activities of caspases were measured in control and MGP-treated activated HSCs. As depicted in Fig. 4D, MGP-induced apoptosis of activated HSCs was associated with increased activities of caspase-2, caspase-3, caspase-8, and caspase-9. Moreover, selective inhibitors of caspase-2 (Z-VDVAD-fmk), caspase-3 (Z-DEVD-fmk), caspase-8 (Z-IETD-fmk), and caspase-9 (Z-LEHD-fmk) could effectively attenuate MGP-triggered apoptosis of activated HSCs (Fig. 4E), suggesting that MGP induced caspase-dependent apoptotic cell death in activated HSCs. Since MGP induced both intrinsic and extrinsic caspase activation, the regulation of the Bcl-2 family and molecules in the death receptor family by MGP was examined. As shown in Fig. 4F, treatment with MGP significantly decreased the levels of anti-apoptotic proteins, Bcl-2 and Mcl-1. In contrast, the proapoptotic molecules, Bax and Fas, were increased in MGP-treated HSCs.

### MGP Suppresses LPS-stimulated Cytokine Production in Kupffer Cells

**Figure 5:**
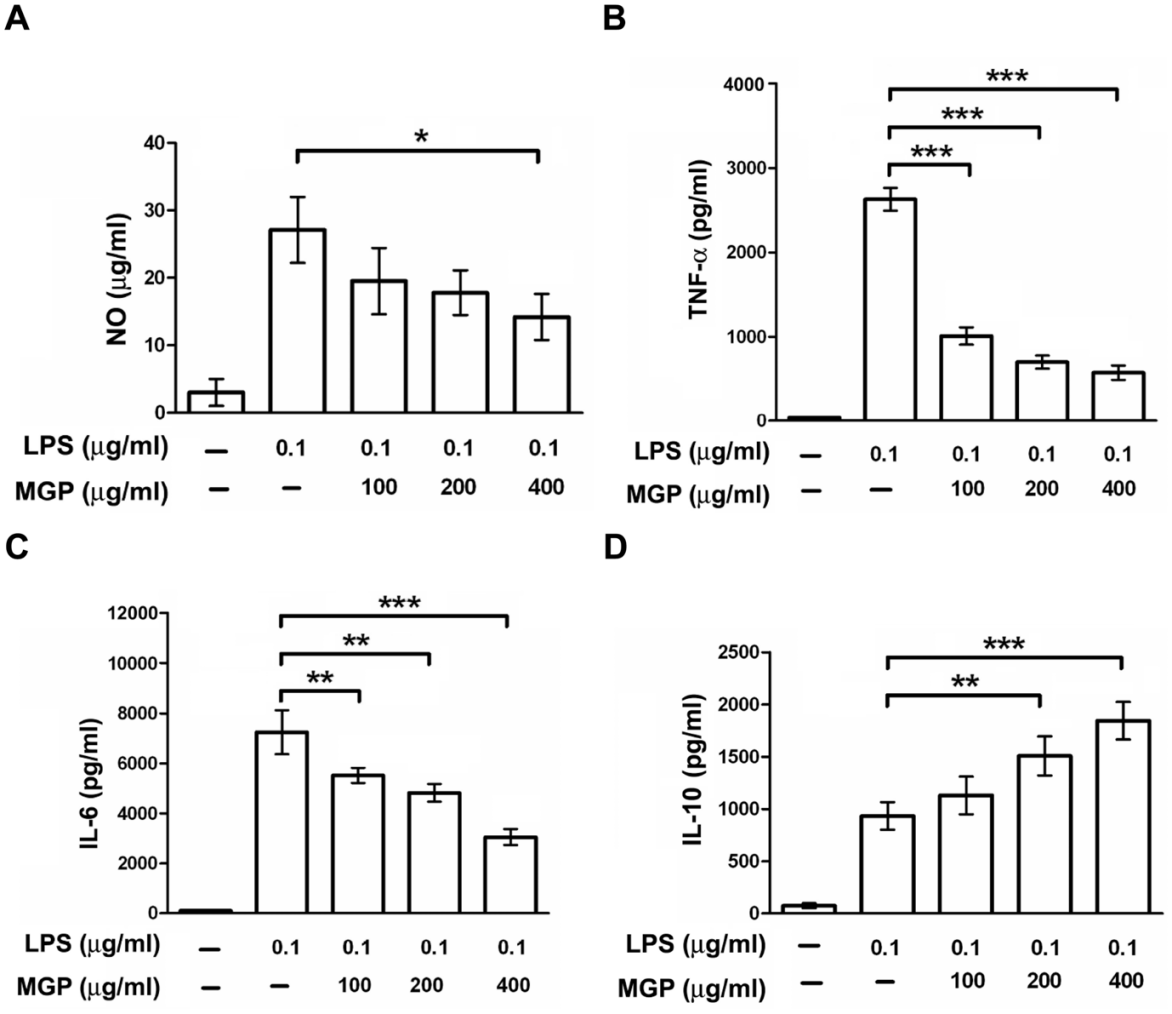
MGP reduced LPS-stimulated inflammatory cytokines and NO production. Cultured rat Kupffer cells were pretreated with MGP (100, 200, and 400 µg/ml) for 30 min and then stimulated with LPS (0.1 µg/ml). Culture media were collected at 24 h for (**A**) NO, (**B**) TNF-α, and (**C**) IL-6 analysis, and at 48 h for (**D**) IL-10 measurement. Each value is presented as the mean±S.D. from three independent experiments. Significance for *p*-values<0.05, 0.001 and 0.0001 is marked with *, ** and ***, respectively.

Accumulating evidence indicates that the bulk release of inflammatory mediators by Kupffer cells is critical during the early stages of liver inflammation and fibrosis [23]. Both experimental animal studies and clinical investigations have suggested that the endogenous gut-derived endotoxin LPS is an important cofactor in the pathogenesis of liver injury and fibrosis [24], [25], [26], [27]. Therefore, the effects of MGP on LPS-stimulated cytokines and NO production in primary cultured rat Kupffer cells were characterized. The basic levels of TNF-α, IL-6, IL-10, and NO production in rat Kupffer cells were low. However, following 0.1 µg/ml LPS administration, the production of these cytokines in Kupffer cells significantly increased. Treatment with MGP decreased LPS-stimulated NO (Fig. 5A), TNF-α (Fig. 5B), and IL-6 (Fig. 5C) production in a dose-dependent manner. MGP also exerted its anti-inflammatory action through the induction of IL-10 (Fig. 5D). These results suggest that MGP might suppress liver inflammation and fibrogenesis partly by inhibiting Kupffer cell activation.

### MGP Attenuates the CCl_4_-induced Acute Hepatic Damage and Inflammation *in vivo*

**Figure 6:**
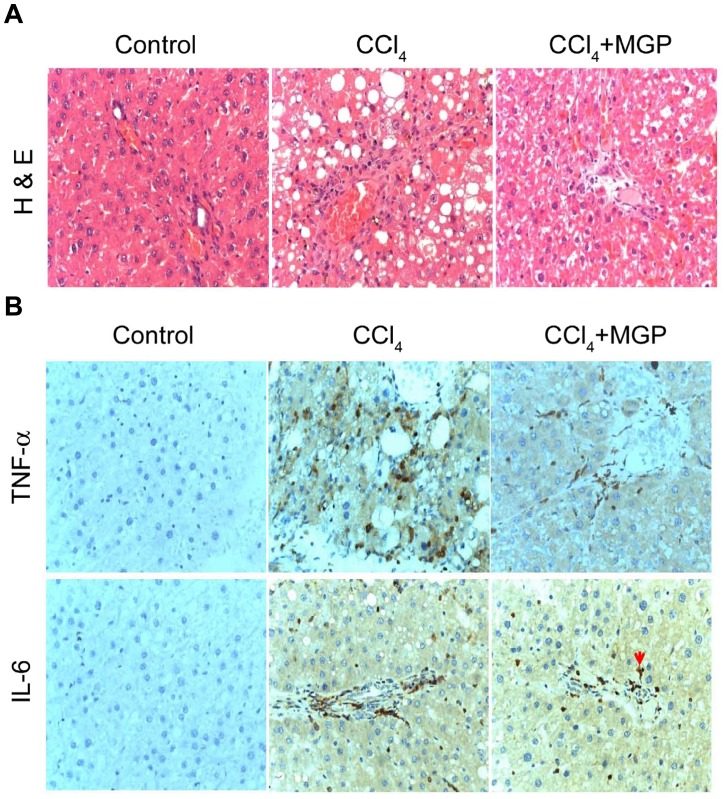
Protective effects of MGP against CCl_4_ induced acute liver inflammation. SD rats were intraperitoneally injected with 2 ml/kg CCl_4_ to induce acute hepatotoxicity and were orally fed with 400 mg/kg MGP daily, rats were sacrificed after 4 days. There were three experimental groups of rat (n = 6 per group), namely: control rat (vehicle only), CCl_4_-treated rat and CCl_4_+MGP treated rat. Liver sections were prepared, and HE staining and immunostaining were performed. (A) H&E staining. CCl_4_ treatment caused extensive necrosis and inflammation; MGP+CCl_4_ treatment caused slight inflammation in the rat livers. However, no significant necrosis and inflammation were observed in control. (B) Histoimmunostaining. Rat liver sections were immunostained with anti-TNF-α and IL-6 antibodies. CCl_4_ treatment markedly increased the expressed levels of TNF-α and IL-6 in rat liver; however, MGP+CCl_4_ treatment significantly reduced the levels of TNF-α and IL-6.

The inflammation usually appears to improve fibrosis formation, so we further examined histopathology or immunohistochemistry in a CCl_4_ induced acute hepatotoxic model. We found that intraperitoneal administration of CCl_4_ resulted in markedly greater histological evidence of necrosis and inflammation compared to control group. In contrast, oral feeding with MGP blunted Kupffer cell and leukocyte accumulation in necrotic areas (Fig. 6A). Moreover, MGP also effectively reduced the CCl_4_-stimulated TNF-α and IL-6 expression (Fig. 6B).

## Discussion

Chinese herbal medicines have been used over thousands of years on the basis of clinical experience and practice, but the underlying mechanisms in most of these medications have not been characterized. We demonstrated here that the methanolic extract of GP, a hepatoprotective herbal medicine that is used in Taiwan, ameliorated injury and fibrosis in liver caused by toxic chemical (DMN and CCl_4_) administration in rats. In cellular studies, MGP prevented activation of transformation and suppressed α-SMA and collagen I expression in primary cultured HSCs. In addition, MGP treatment inhibited cell growth and induced apoptosis in activated HSCs. Moreover, LPS-stimulated inflammatory cytokine production in Kupffer cells was attenuated by MGP. We thus hypothesized that MGP may suppress the activation of HSCs and Kupffer cells during the course of liver inflammation and fibrosis, alleviating subsequent liver injury and fibrosis progression.

Accumulating evidence indicates that activated HSC plays a crucial role in the development and resolution of liver fibrosis [28]. Therefore inhibiting activation of HSCs and stimulating their apoptosis could be an effective treatment for liver fibrosis. Here, we found that MGP effectively inhibited the myofibroblast-like activation of isolated rat HSCs and reduced the expression of α-SMA and collagen I *in vitro*. In addition, treatment with MGP caused a mitochondrial-dependent apoptosis in activated HSCs. Therefore, suppression of myofibroblast-like transformation and induction of apoptotic death in activated HSCs upon MGP treatment may help alleviate chemical (both DMN and CCl_4_)-induced liver fibrosis *in vivo*. Inflammation is essential for host defense during liver injury, but an uncontrolled inflammatory response is the common mechanism involved in the majority of clinical liver pathologies, resulting in tissue damage, fibrosis, and cirrhosis [29]. Kupffer cells, the resident hepatic macrophages, play a crucial role in liver inflammation and fibrogenesis [30]. In acute or chronic liver diseases, Kupffer cells and infiltrating monocyte-derived macrophages are well known to promote inflammatory cascades by releasing these pro-inflammatory mediators with consequences such as T cell attraction, induction of hepatocyte apoptosis, and activation of fibrogenic HSCs [29], [31]. Therefore, depletion of Kupffer cell function significantly alleviates thioacetamide-induced hepatotoxicity [32]. In addition, selective inhibition of Kupffer cells by administration of either gadolinium chloride or methyl palmitate results in abrogation of liver injury [30], [33], [34]. Therefore, the factors that control the Kupffer cell response are clearly critical in the progression of liver fibrosis. In this study, MGP exhibited strong anti-inflammatory activity by reducing the production of TNF-α, IL-6 and NO in LPS-stimulated Kupffer cells (Figs. 6A, 6B, and 6C), suggesting that inhibition of proinflammatory cytokines (TNF-α and IL-6) and no production by kupffer cells may contribute to MGP-mediated liver protection.

Increases in anti-inflammatory mediators also may contribute to an anti-inflammatory state in the liver and ameliorate injury. For example, IL-10, an immunomodulatory cytokine with potent anti-inflammatory and immunosuppressive properties, decreases production of pro-inflammatory cytokines, including TNF-α and IL-1β [35]. The ability of IL-10 to modulate the inflammatory response and to limit hepatotoxicity has been shown in several models of liver injury [36], [37], [38]. Previous reports have indicated that IL-10 has a role in the remodeling of the extracellular matrix. *In vitro*, IL-10 downregulates collagen I and upregulates metalloproteinase gene expression [39]. IL-10 might also have antifibrogenic properties through downregulation of profibrogenic cytokines, like TGF-β1 [40]. Increased endogenous IL-10 expression ameliorates acute inflammatory burst and subsequent liver fibrosis after repeated stimulations with CCl_4_ [41]. Endogenous IL-10 synthesized during the course of liver inflammation and fibrosis may modulate Kupffer cell actions, and influence the subsequent progression of fibrosis [42]. Our results also showed that MGP effectively increased IL-10 generation in isolated rat Kupffer cells. Taken together, it is likely that MGP suppressed the activation of Kupffer cells and modulated the expression of cytokines during DMN or CCl_4_ intoxication, thereby decreasing inflammation and the subsequent fibrogenic response in the liver.

In this study, we found that MGP was safe and nontoxic for *in vitro* and *in vivo* experimental use. Cellular studies demonstrated that MGP had no cytotoxic effect on primary cultured hepatocytes, as examined by morphological investigation and viable cell counting. In addition, our pilot study for MGP toxicity indicated that daily oral administration of a high dose of MGP (1.6 g/kg body weight) for nine months did not cause any apparent adverse side-effects in rats, and there was no evidence of mortality or hepatic or renal injury in histological sections of liver and kidney (data not shown). Moreover, these MGP-fed rats did not exhibit any significant changes in body weight, liver weight, or serum ALT, AST, bilirubin, albumin, BUN, and creatinine levels in comparison with the control group (data not shown), suggesting that MGP had no liver or renal toxicity at the tested doses. In Taiwan, the daily intake amount suggested by folk medicine for treatment of chronic liver disorder patients is 200 g/day of fresh GP, this dosage is equal to 55 mg/kg/day of MGP. In our animal study, DMN or CCl_4_-injured rats were fed with 400 mg/kg/day of MGP. We translated this dosage from rat to human by the Meeh-Rubner formula and obtained an equivalent human intake amount of 60 mg/kg/day of MGP. These data demonstrate that the daily intake amount suggested by folk medicine is extremely consistent with the translated amount of our animal study (55 mg/kg/day *v.s.* 60 mg/kg/day). Further *in vivo* and *in vitro* studies, including the use of different cell types in the liver (e.g., HSCs, Kupffer cells and sinusoidal endothelial cells), will be required to determine the exact molecular pathways affected by MGP, which may be useful in the development of novel treatments for hepatic injury and fibrosis.

In conclusion, GP is a folk herbal medicine with hepatoprotective effects that is used in Taiwan. Our findings showed that the 70% methanol extract of GP prevented fibrogenesis responses induced by toxic chemicals (DMN and CCl_4_) *in vivo*. In cellular studies, GP extract exhibited anti-inflammatory activity via suppressing LPS-stimulated pro-inflammatory cytokines and mediator production in liver macrophage lineage Kupffer cells. Moreover, GP extract effectively suppressed activation of HSC transformation, inhibited cell growth, and induced apoptosis in activated HSCs. Our findings confirm that GP extract has hepatoprotective and anti-fibrotic activities and that it has the potential to become a novel and promising antifibrotic medicine for liver fibrosis. The modes of action and the active components of GP extract require further elucidation.

## References

[1] Poli G (2000) Pathogenesis of liver fibrosis: role of oxidative stress. Mol Aspects Med 21: 49–98. 10978499 10.1016/s0098-2997(00)00004-2

[2] Brenner DA (2009) Molecular pathogenesis of liver fibrosis. Trans Am Clin Climatol Assoc 120: 361–368. 19768189 PMC2744540

[3] Rosenbloom J, Castro SV, Jimenez SA Narrative review: fibrotic diseases: cellular and molecular mechanisms and novel therapies. Ann Intern Med 152: 159–166. 20124232 10.7326/0003-4819-152-3-201002020-00007

[4] Lissoos TW, Beno DW, Davis BH (1992) Hepatic fibrogenesis and its modulation by growth factors. J Pediatr Gastroenterol Nutr 15: 225–231. 1432458 10.1097/00005176-199210000-00001

[5] Friedman SL (1993) Seminars in medicine of the Beth Israel Hospital, Boston. The cellular basis of hepatic fibrosis. Mechanisms and treatment strategies. N Engl J Med 328: 1828–1835. 8502273 10.1056/NEJM199306243282508

[6] Mavier P, Mallat A (1995) Perspectives in the treatment of liver fibrosis. J Hepatol 22: 111–115. 7665845

[7] Kaplowitz N (1997) Hepatotoxicity of herbal remedies: insights into the intricacies of plant-animal warfare and cell death. Gastroenterology 113: 1408–1412. 9322538 10.1053/gast.1997.v113.agast971131408

[8] Chung YC, Chen SJ, Hsu CK, Chang CT, Chou ST (2005) Studies on the antioxidative activity of Graptopetalum paraguayense E. Walther. Food Chemistry 91: 5.

[9] Chen SJ, Chung JG, Chung YC, Chou ST (2008) In vitro antioxidant and antiproliferative activity of the stem extracts from Graptopetalum paraguayense. Am J Chin Med 36: 369–383. 18457367 10.1142/S0192415X08005837

[10] Chen SJ, Chang CT, Chung YC, Chou ST (2007) Studies on the inhibitory effect of Graptopetalum paraguayense E. Walther extracts on the angiotensin converting enzyme. Food Chemistry 100: 5.

[11] Huang B, Ban X, He J, Zeng H, Zhang P, et al (2010) Hepatoprotective and antioxidant effects of the methanolic extract from Halenia elliptica. J Ethnopharmacol 131: 276–281. 20600758 10.1016/j.jep.2010.06.029

[12] Pines M, Knopov V, Genina O, Lavelin I, Nagler A (1997) Halofuginone, a specific inhibitor of collagen type I synthesis, prevents dimethylnitrosamine-induced liver cirrhosis. J Hepatol 27: 391–398. 9288615 10.1016/s0168-8278(97)80186-9

[13] Lin JM, Lin CC, Chiu HF, Yang JJ, Lee SG (1993) Evaluation of the anti-inflammatory and liver-protective effects of anoectochilus formosanus, ganoderma lucidum and gynostemma pentaphyllum in rats. Am J Chin Med 21: 59–69. 8328423 10.1142/S0192415X9300008X

[14] Knodell RG, Ishak KG, Black WC, Chen TS, Craig R, et al (1981) Formulation and application of a numerical scoring system for assessing histological activity in asymptomatic chronic active hepatitis. Hepatology 1: 431–435. 7308988 10.1002/hep.1840010511

[15] Ishak K, Baptista A, Bianchi L, Callea F, De Groote J, et al (1995) Histological grading and staging of chronic hepatitis. J Hepatol 22: 696–699. 7560864 10.1016/0168-8278(95)80226-6

[16] Su LJ, Hsu SL, Yang JS, Tseng HH, Huang SF, et al (2006) Global gene expression profiling of dimethylnitrosamine-induced liver fibrosis: from pathological and biochemical data to microarray analysis. Gene Expr 13: 107–132. 17017125 10.3727/000000006783991872 PMC6032472

[17] Gascon-Barre M, Huet PM, Belgiorno J, Plourde V, Coulombe PA (1989) Estimation of collagen content of liver specimens. Variation among animals and among hepatic lobes in cirrhotic rats. J Histochem Cytochem 37: 377–381. 2465335 10.1177/37.3.2465335

[18] Rojkind M, Gonzalez E (1974) An improved method for determining specific radioactivities of proline-14C and hydroxyproline-14C in collagen and in noncollagenous proteins. Anal Biochem 57: 1–7. 4817495 10.1016/0003-2697(74)90043-8

[19] Bataller R, Schwabe RF, Choi YH, Yang L, Paik YH, et al (2003) NADPH oxidase signal transduces angiotensin II in hepatic stellate cells and is critical in hepatic fibrosis. J Clin Invest 112: 1383–1394. 14597764 10.1172/JCI18212 PMC228420

[20] Gandhi CR, Hanahan DJ, Olson MS (1990) Two distinct pathways of platelet-activating factor-induced hydrolysis of phosphoinositides in primary cultures of rat Kupffer cells. J Biol Chem 265: 18234–18241. 2170406

[21] Recknagel RO, Glende EA Jr, Dolak JA, Waller RL (1989) Mechanisms of carbon tetrachloride toxicity. Pharmacol Ther 43: 139–154. 2675128 10.1016/0163-7258(89)90050-8

[22] Jezequel AM, Mancini R, Rinaldesi ML, Macarri G, Venturini C, et al (1987) A morphological study of the early stages of hepatic fibrosis induced by low doses of dimethylnitrosamine in the rat. J Hepatol 5: 174–181. 3693862 10.1016/s0168-8278(87)80570-6

[23] Heymann F, Trautwein C, Tacke F (2009) Monocytes and macrophages as cellular targets in liver fibrosis. Inflamm Allergy Drug Targets 8: 307–318. 19534673 10.2174/187152809789352230

[24] Su GL (2002) Lipopolysaccharides in liver injury: molecular mechanisms of Kupffer cell activation. Am J Physiol Gastrointest Liver Physiol 283: G256–265. 12121871 10.1152/ajpgi.00550.2001

[25] Enomoto N, Ikejima K, Bradford B, Rivera C, Kono H, et al (1998) Alcohol causes both tolerance and sensitization of rat Kupffer cells via mechanisms dependent on endotoxin. Gastroenterology 115: 443–451. 9679050 10.1016/s0016-5085(98)70211-2

[26] Suzuki S, Nakamura S, Serizawa A, Sakaguchi T, Konno H, et al (1996) Role of Kupffer cells and the spleen in modulation of endotoxin-induced liver injury after partial hepatectomy. Hepatology 24: 219–225. 8707266 10.1053/jhep.1996.v24.pm0008707266

[27] Enomoto N, Ikejima K, Yamashina S, Hirose M, Shimizu H, et al (2001) Kupffer cell sensitization by alcohol involves increased permeability to gut-derived endotoxin. Alcohol Clin Exp Res 25: 51S–54S. 11410742 10.1097/00000374-200106001-00012

[28] Kisseleva T, Brenner DA (2007) Role of hepatic stellate cells in fibrogenesis and the reversal of fibrosis. J Gastroenterol Hepatol 22 Suppl 1S73–78. 17567473 10.1111/j.1440-1746.2006.04658.x

[29] Tacke F, Luedde T, Trautwein C (2009) Inflammatory pathways in liver homeostasis and liver injury. Clin Rev Allergy Immunol 36: 4–12. 18600481 10.1007/s12016-008-8091-0

[30] Winwood PJ, Arthur MJ (1993) Kupffer cells: their activation and role in animal models of liver injury and human liver disease. Semin Liver Dis 13: 50–59. 8446908 10.1055/s-2007-1007337

[31] Bilzer M, Roggel F, Gerbes AL (2006) Role of Kupffer cells in host defense and liver disease. Liver Int 26: 1175–1186. 17105582 10.1111/j.1478-3231.2006.01342.x

[32] Andres D, Sanchez-Reus I, Bautista M, Cascales M (2003) Depletion of Kupffer cell function by gadolinium chloride attenuates thioacetamide-induced hepatotoxicity. Expression of metallothionein and HSP70. Biochem Pharmacol 66: 917–926. 12963478 10.1016/s0006-2952(03)00443-x

[33] Adachi Y, Bradford BU, Gao W, Bojes HK, Thurman RG (1994) Inactivation of Kupffer cells prevents early alcohol-induced liver injury. Hepatology 20: 453–460. 8045507

[34] Laskin DL (1990) Nonparenchymal cells and hepatotoxicity. Semin Liver Dis 10: 293–304. 2281337 10.1055/s-2008-1040485

[35] O’Shea JJ, Murray PJ (2008) Cytokine signaling modules in inflammatory responses. Immunity 28: 477–487. 18400190 10.1016/j.immuni.2008.03.002 PMC2782488

[36] Louis H, Le Moine O, Peny MO, Quertinmont E, Fokan D, et al (1997) Production and role of interleukin-10 in concanavalin A-induced hepatitis in mice. Hepatology 25: 1382–1389. 9185757 10.1002/hep.510250614

[37] Louis H, Le Moine O, Peny MO, Gulbis B, Nisol F, et al (1997) Hepatoprotective role of interleukin 10 in galactosamine/lipopolysaccharide mouse liver injury. Gastroenterology 112: 935–942. 9041256 10.1053/gast.1997.v112.pm9041256

[38] Arai T, Hiromatsu K, Kobayashi N, Takano M, Ishida H, et al (1995) IL-10 is involved in the protective effect of dibutyryl cyclic adenosine monophosphate on endotoxin-induced inflammatory liver injury. J Immunol 155: 5743–5749. 7499862

[39] Reitamo S, Remitz A, Tamai K, Uitto J (1994) Interleukin-10 modulates type I collagen and matrix metalloprotease gene expression in cultured human skin fibroblasts. J Clin Invest 94: 2489–2492. 7989607 10.1172/JCI117618 PMC330082

[40] Van Vlasselaer P, Borremans B, van Gorp U, Dasch JR, De Waal-Malefyt R (1994) Interleukin 10 inhibits transforming growth factor-beta (TGF-beta) synthesis required for osteogenic commitment of mouse bone marrow cells. J Cell Biol 124: 569–577. 8106554 10.1083/jcb.124.4.569 PMC2119922

[41] Louis H, Van Laethem JL, Wu W, Quertinmont E, Degraef C, et al (1998) Interleukin-10 controls neutrophilic infiltration, hepatocyte proliferation, and liver fibrosis induced by carbon tetrachloride in mice. Hepatology 28: 1607–1615. 9828225 10.1002/hep.510280621

[42] Thompson K, Maltby J, Fallowfield J, McAulay M, Millward-Sadler H, et al (1998) Interleukin-10 expression and function in experimental murine liver inflammation and fibrosis. Hepatology 28: 1597–1606. 9828224 10.1002/hep.510280620

